# Supplementary material for: The Role of Positron Emission Tomography in Clinical Management of Intraductal Papillary Mucinous Neoplasms of the Pancreas
Source: Cancers (Basel). 2020 Mar 27;12(4):807. doi: 10.3390/cancers12040807 (PMC7226258; doi:10.3390/cancers12040807)
Supplement: Supplementary file 1 [file cancers-12-00807-s001.pdf]

Supplementary Materials: The Role of Positron Emission Tomography in Clinical Management of Intraductal Papillary Mucinous Neoplasms of the Pancreas.

Simone Serafini, Cosimo Sperti, Alessandra Rosalba Brazzale, Diego Cecchin, Pietro Zucchetta, Elisa Sefora Pierobon, Alberto Ponzoni, Michele Valmasoni and Lucia Moletta

Text S1. Report of statistical analysis performed for two sample binary diagnostic tests. 18-FDG PET (Test1) and ICG (Test 2).

Counts for Tests 1 and 2

	**Counts for Test 1**			**Counts for Test 2**		
**True**	**Diagnostic Test Result**			**Diagnostic Test Result**		
**Condition**	**Positive**	**Negative**	**Total**	**Positive**	**Negative**	**Total**
Present	168	43	211	152	74	226
Absent	10	198	208	78	108	186
Total	178	241	419	230	182	412

Two Sample Binary Diagnostic Tests

Sensitivity Confidence Intervals Section

**Statistic**	**Test**	**Value**	**Lower 95.0% Conf. Limit**	**Upper 95.0% Conf. Limit**
Sensitivity (Se1)	1	0.7962	0.7368	0.8450
Sensitivity (Se2)	2	0.6726	0.6089	0.7304
Difference (Se1-Se2)		0.1236	0.0411	0.2050
Ratio (Se1/Se2)		1.1838	1.0575	1.3305

Specificity Confidence Intervals Section

**Statistic**	**Test**	**Value**	**Lower 95.0% Conf. Limit**	**Upper 95.0% Conf. Limit**
Specificity (Sp1)	1	0.9519	0.9138	0.9737
Specificity (Sp2)	2	0.5806	0.5088	0.6492
Difference (Sp1-Sp2)		0.3713	0.2948	0.4480
Ratio (Sp1/Sp2)		1.6394	1.4572	1.8763

Likelihood Ratio Section

**Statistic**	**Test**	**Value**	**Lower 95.0% Conf. Limit**	**Upper 95.0% Conf. Limit**
LR(Test=Posivitive)	1	16.5611	9.4512	32.2283
	2	1.6038	1.3319	1.9587
LR(Test=Negative)	1	0.2141	0.1611	0.2759
	2	0.5639	0.4486	0.7024

Odds Ratio Section

			**Lower 95.0%**	**Upper 95.0%**

Statistic	Test	Value	Conf. Limit	Conf. Limit
Odds Ratio (+ 1/2)	1	73.2288	36.2013	148.1290
	2	2.8293	1.8939	4.2265
Odds Ratio (Fleiss)	1	73.2288	36.0892	170.3492
	2	2.8293	1.8643	4.3435

Hypothesis Tests of the Equivalence of Sensitivity

Statistic	Prob Level	Lower 90.0% Conf. Limit	Upper 90.0% Conf. Limit	Lower Equiv. Bound	Upper Equiv. Bound	Reject H0 and Conclude Equivalence at the 5.0% Significance Level
Diff. (Se1-Se2)	0.0012	0.0545	0.1920	-0.2500	0.2500	Yes
Ratio (Se1/Se2)	0.1770	1.0769	1.3051	0.8000	1.2500	No

Notes:
Equivalence is concluded when the confidence limits fall completely inside the equivalence bounds.

Hypothesis Tests of the Equivalence of Specificity

Statistic	Prob Level	Lower 90.0% Conf. Limit	Upper 90.0% Conf. Limit	Lower Equiv. Bound	Upper Equiv. Bound	Reject H0 and Conclude Equivalence at the 5.0% Significance Level
Diff. (Sp1-Sp2)		0.3071	0.4356	-0.2500	0.2500	No
Ratio (Sp1/Sp2)		1.4837	1.8337	0.8000	1.2500	No

Notes:
Equivalence is concluded when the confidence limits fall completely inside the equivalence bounds.

Tests Showing the Sensitivity Non-inferiority of Test 1 Compared to Test 2

Statistic	Prob Level	Lower 90.0% Conf. Limit	Upper 90.0% Conf. Limit	Lower Equiv. Bound	Upper Equiv. Bound	Reject H0 and Conclude Non-inferiority at the 5.0% Significance Level
Diff. (Se1-Se2)	0.0000	0.0545	0.1920	-0.2500	0.2500	Yes
Ratio (Se1/Se2)	0.0000	1.0769	1.3051	0.8000	1.2500	Yes

Notes:
H0: The sensitivity of Test 1 is inferior to Test 2.
Ha: The sensitivity of Test 1 is non-inferior to Test 2.

Tests Showing the Specificity Non-inferiority of Test 1 Compared to Test 2

Statistic	Prob Level	Lower 90.0% Conf. Limit	Upper 90.0% Conf. Limit	Lower Equiv. Bound	Upper Equiv. Bound	Reject H0 and Conclude Non-inferiority at the 5.0% Significance Level
Diff. (Sp1-Sp2)		0.3071	0.4356	-0.2500	0.2500	Yes
Ratio (Sp1/Sp2)		1.4837	1.8337	0.8000	1.2500	Yes

Notes:
H0: The specificity of Test 1 is inferior to Test 2.

Ha: The specificity of Test 1 is non-inferior to Test 2.

-- Confidence Interval Method

Difference C.I. Method:	Score w/ Skewness (Gart-Nam)
Ratio C.I. Method:	Score w/ Skewness (Gart-Nam)

-- Report Options

Alpha - C.I.'s:	0.05
Alpha - Tests:	0.05
Proportion Decimals:	4
Probability Decimals:	4

-- Equivalence or Non-Inferiority Settings

Max Equivalence Difference:	0.25
Max Equivalence Ratio:	1.25

© 2020 by the authors. Licensee MDPI, Basel, Switzerland. This article is an open access article distributed under the terms and conditions of the Creative Commons Attribution (CC BY) license (http://creativecommons.org/licenses/by/4.0/).
